# Media Matters, Examining Historical and Modern *Streptococcus pneumoniae* Growth Media and the Experiments They Affect

**DOI:** 10.3389/fcimb.2021.613623

**Published:** 2021-03-23

**Authors:** Yamil Sanchez-Rosario, Michael D. L. Johnson

**Affiliations:** ^1^ Department of Immunobiology, University of Arizona, Tucson, AZ, United States; ^2^ BIO5 Institute, University of Arizona, Tucson, AZ, United States; ^3^ Valley Fever Center for Excellence, University of Arizona, Tucson, AZ, United States

**Keywords:** *Streptococcus pneumoniae* (pneumococcus), media, metabolism, growth, experimental rigor

## Abstract

While some bacteria can thrive for generations in minerals and salts, many require lavish nutrition and specific chemicals to survive to the point where they can be observed and researched. Although researchers once boiled and rendered animal flesh and bones to obtain a media that facilitated bacterial growth, we now have a plethora of formulations and manufacturers to provide dehydrated flavors of historical, modified, and modern media. The purpose of media has evolved from simple isolation to more measured study. However, in some instances, media formulated to aid the metabolic, nutritional, or physical properties of microbes may not be best suited for studying pathogen behavior or resilience as a function of host interactions. While there have been comparative studies on handfuls of these media in *Streptococcus pneumoniae*, this review focuses on describing both the historical and modern composition of common complex (Todd Hewitt and M17), semi-defined (Adams and Roe), and defined pneumococcal media (RPMI and Van de Rijn and Kessler), key components discovered/needed for cultivation/growth enhancement, and effects these different media have on bacterial phenotypes and experimental outcomes. While many researchers find the best conditions to grow and experiment on their bacteria of choice, the reasons for some researchers to use a specific medium is at best, not discussed, and at worst, arbitrary. As such, the goal of this review is to highlight the differences in pneumococcal media to encourage investigators to challenge their decisions on why they use a given medium, discuss the recipe, and explain their reasoning.

## Isolation Media for Pneumococcus in the 19^th^ Century

*Streptococcus pneumoniae*, or the pneumococcus, was fortuitously discovered on two continents simultaneously. In France, Louis Pasteur was studying the etiology of rabies, and in North America, George M. Sternberg was studying malaria. In North America, the future Surgeon General Sternberg inoculated two rabbits with his saliva to test whether an innocuous substance would produce an immune response. To his amazement, both rabbits died from a cause of death distinct from malaria (Sternberg, 1881). Sternberg inoculated additional rabbits with his saliva as well as dogs, chickens, guinea pigs, and rats. This led to septicemia in the rabbits and guinea pigs, but not in the dogs or chickens. However, 1 ml of serum from a recently diseased rabbit put into a dog proved fatal (Sternberg, 1881). Three months later, Pasteur investigated a hospitalized child in the later stages of rabies to procure a saliva sample. When the sample was later inoculated into rabbits two days later, the rabbits died, but not from rabies. Upon microscopic examination, Pasteur observed an “8” shaped microorganism with a halo surrounding it and was only successful in obtaining cultures in veal broth (Pasteur, 1881).

Sternberg encouraged other investigators to test their saliva and later learned that saliva from some, but not all, led to death of the rabbits. This result created speculation about the nature of sepsis and whether it was attributed to putrefaction (decaying of organic matter) (Sternberg, 1881; Talamon et al., 1883). Other types of putrid inoculums, such as sewage, could produce sepsis. However, this, at the time, was mostly attributed to bacilli (Sternberg, 1881). Sternberg found that incubating saliva to the point of putrefaction (usually 24 h at 37˚ C) curtailed its lethality. Further, attempts to isolate the organism (which he would later call *Micrococcus pasteuri*) straight from saliva into artificial media proved impossible. It is unknown whether this was due to the nature of these media, or what we know now to be the bacterium’s inherent autolytic ability. He tried cultivating the organism in urine, gelatin, and dog bouillon (a broth or extract made by stewing the subject in water) unsuccessfully; however, similarly to Pasteur, if the inoculum was amplified in rabbit’s blood, then it would grow on rabbit flesh bouillon (Sternberg, 1881). A bacteriology manual from 1896 written by Sternberg explains bouillon preparation as ~500 g of chopped meat per liter of water, cooked for 30 minutes in a glass flask or enameled iron kettle, then filtered, neutralized, cooked again to coagulate and remove albuminoids, filtered, and finally sterilized (Sternberg, 1896). Others from the era utilize a different procedure; half a kg of chopped meat is soaked in 1 L of cold water for 24 hours, strained to the same volume, combined with 1% peptone w/w (partial enzymatic digestion or acid hydrolysis of yeast, animal, or plant tissues), and 0.5% NaCl w/w (Stedman, 1900). The mixture was then alkalinized by addition of sodium carbonate, heated to coagulate and precipitate albumins, filtered, and finally sterilized by autoclave.

In November 1883, French physician Charles Talamon was the first to publish the isolation of a pure culture of the pneumococcus (*Coccus lanceolatus*) (Talamon et al., 1883). He described taking his inoculum from the blood of rabbits and culturing it in “bouillon de Liebig,” from Liebig’s Extract of Meat Company which was famous in the late 1800s for making a variety of products from meat sources (Cansler, 2013). The preparation of bouillon de Liebig included peptone plus sodium chloride, in combination with meat (by record, most likely beef) infusion (the product of allowing the meat to stay in a solvent for a prolonged period of time), and then alkalizing by either adding NaHCO_3_ directly or passing the medium through a carbonate column. The addition of the infusion or extract supplemented the peptone by adding minerals, phosphates, and vitamins. This medium differed from those prepared in labs by skipping the coagulation and removal of albumin from the meat infusion. However, it is now known that albumin can actually promote pneumococcal growth at low concentrations (Ruiz et al., 2011).

In 1892, William H. Welch published a research article describing the history of the pneumococcus (*Micrococcus lanceolatus*), as well as many characteristics of the organism (Welch, 1892). He described the pneumococcus’ preference for alkaline media and its ability to grow on solid media such as nutrient agar, which is 5 g Liebig bouillon, 30 g peptone, 5 g cane sugar (sucrose), and 15 g agar, and “glycerine agar,” which is nutrient agar + 5% glycerine/glycerol. In his communication, Welch mentioned his preference for a modification of Guarnieri’s formulation which contains per liter 950 g of meat infusion, 5 g of NaCl, 10 g of peptone, 6-8 g agar, and 30-40 g of gelatin (a product of cooking collagen) with slightly alkaline pH (Welch, 1892).

## Sources of Variability in Media Components

Variability in media was a major problem during earlier investigations on *S. pneumoniae*. Media were composed of a few undefined components and prepared by different methods. Different meat sources, qualities, and preparation (soaked vs. cooked) greatly affected the nutritional components of the media. As such, Welch and others had challenges in obtaining quality peptone capable of sustaining pneumococcal growth, even within the same brand (Fennel and Fisher, 1919). Peptones supply a source of amino acids and nitrogen for bacterial growth. However, because the raw input can be sourced from plants (soy), animals (milk, fetal sera) and microbes (yeast), their amino acid profiles are diverse. There are several methods used to process raw protein sources. For example, peptones derived from albumins are subjected to gastric and pancreatic digestion, acid hydrolysis, heating, and precipitation (Zimbro and Power, 2009). Thermal degradation of bulk protein can convert amino acids such as tryptophan to indoles or phenols (Kato et al., 1971). During acid hydrolysis, tryptophan can be completely lost, cystine can be partially broken down, and labile vitamins are mostly destroyed (Zimbro and Power, 2009). Enzymatic digest is gentler, but each proteolytic enzyme has a distinct cleavage pattern, which leads to the diverse array of molecular weight products. Finally, all of these changes take place *before* the media are autoclaved.

Another nuisance involving peptones is their ability to retain carbohydrate contaminants. In 1950, *The American Journal of Public Health* conducted a survey that evaluated 450 samples of peptones and 60 samples of bacteriological culture media for the presence of fermentable carbohydrates (Vera, 1950). The samples were tested against several bacteria including *Streptococcal* spp. The study concluded that all peptone sample sources, and most meat sources were not suitable for bacterial fermentation tests as they still contained fermentable substances detected *via* acid or acid gas production. Recent documentation provided by different manufacturers also indicates the presence of carbohydrates in peptones from diverse origins (Klotz et al., 2017). This observation was significant, given that large segments of pneumococcal DNA are devoted to carbohydrate metabolism and transport (Bidossi et al., 2012). Additional work by Troxler et al. demonstrated that choice of carbon source had an impact on capsule expression and growth as sugars like fructose are not converted into capsule precursors thus limiting capsule production (Troxler et al., 2019).

Other points of media variability include vitamin and trace metal content. Not surprisingly, the accumulation of vitamins and trace metals vastly differs between animal and plant sources of peptones. For example, while biotin (vitamin B7), nicotinic acid (vitamin B3, nicotinic acid, or niacin is a precursor for nicotinamide adenine dinucleotide), and pantothenic acid (vitamin B5 and necessary for Coenzyme A production) were deemed important vitamins (Rane and Subbarow, 1940; Badger, 1944), Stokes et al. reported that the concentration of these three vitamins varied significantly between different nitrogenous sources (Stokes et al., 1944). This report describes a 60-fold difference in pantothenic acid concentration, 34-fold in nicotinic acid, and 17-fold on biotin among different nitrogen sources.

Trace metals are often important catalytic components for enzymatic reactions, and in doing so, affect gene expression, bacterial growth, and development. They can also be toxic in excess. As such, a bacterium’s ability to maintain metal homeostasis is essential for survival. However, historically, there have been discrepancies in trace metal concentrations (Grant and Pramer, 1962; Nolan and Nolan, 1972). A modern analysis reports 2-fold differences in zinc and iron concentrations, and a 40-fold difference for copper between yeast extracts and casein or meat sources (Klotz et al., 2017). In high concentrations, copper has been shown to be toxic to pneumococcal growth by inhibiting nucleotide synthesis (Johnson et al., 2015). High zinc concentrations have also shown to be toxic to the pneumococcus by competing with manganese uptake (Eijkelkamp et al., 2014). Elevated exogenous iron has been implicated in enhancing pneumococcal biofilm formation and competence (Trappetti et al., 2011).

Perhaps not part of a specific medium perse, but the culturing environment is also important for growing bacteria. In the 1930’s Gladstone et al. illustrated the importance that CO_2_ can have in the cultivation of other bacteria (Gladstone et al., 1935). CO_2_ greatly improves *S. pneumoniae* growth as specifically demonstrated in 1942 by Kempner and Schlayer (Kempner and Schlayer, 1942). Under ambient CO_2_ concentrations (~300 ppm), *S. pneumoniae* grew in peptone, beef infusion, and glucose broth at higher pH (7.8 vs. 7.4). They postulated that adding CO_2_ should lower the pH and thus, reduce pneumococcal growth, but that the opposite occurred. It is known that the pneumococcus possesses a highly conserved, β-class carbonic anhydrase (turning CO_2_ and water into carbonic acid) that is necessary for growth in ambient air (Smith and Ferry, 2000; Burghout et al., 2010). As such, modern aerobic cultivation of pneumococcal species often requires, or is improved by providing an enriched 5% CO_2_-atmosphere as the benefits of CO_2_ outweighed the resultant media acidity.

Growth media development and use have come full circle. From local raw feedstocks, to gradually refined requirements for optimal cultivation, and back to undefined media for convenience’s sake. However, while most manufacturing processes have been standardized to minimize variance across batches in modern media, they still rely on disparate ingredients for the cultivation of microorganisms. The road to discovering essential nutrients required for pneumococcal growth has been challenging given the fact that so many serotypes exist (100 different capsules define these serotypes) with some being more fastidious than others. While in some cases, the determination of specific ingredients tested a hypothesis, in other cases, it was trial and error to achieve a predetermined goal such as obtaining a pure culture. In the following paragraphs, we introduce some of these media (**Table 1**) and define them to the extent that historical records allow.

**Table 1:** Media Quick Guide.

Medium	Defined or Complex	Short Summary of Findings	Citations
Todd Hewitt	Complex	Used to describe lactic acid formation from glucose breakdown; importance of inorganic phosphate	Todd and Hewitt, 1932 and Hewitt, 1932
Rane and Subarrow	Defined	Defined vitamins, choline, and amino acids necessary for pneumococcal growth of different serotypes	Rane and Subbarow, 1940
Adams and Roe	Semi-defined	Subculture of fastidious serotypes; asparagine crucial for sulfonamide-resistant *Streptococci*	Adams and Roe, 1945
Lacks and Hotchkiss	Semi-defined	Common base for semi-defined media such as C+Y, added catalase as a component	Lacks and Hotchkiss, 1960 and Lacks, 1966
Sicard	Defined	Growth of fastidious serotype (type-2 Avery's strain), vitamin solution used for RPMI to facilitate better growth	Sicard, 1964
M17	Complex	Cultivating lactic acid *Streptococci*, uses metal compatible disodium glycerol-2-phosphat e as buffer	Terzaghi and Sandine, 1975
Van de Rijn and Kessler	Defined	Designed for high yield and short lag to prevent adaptation, mostly for virulence factors, made originally for GAS	Van De Rijn and Kessler, 1980
RPMI 1640	Defined	Common medium for culturing host cells assuring common nutrient aviablity during examine host-pathogen interactions	Sicard, 1964; Brown et al., 2001 and Schulz et al., 2014

## Pneumococcal Media

### Todd Hewitt

Todd Hewitt broth with yeast extract is a popular liquid media used for the cultivation of *S. pneumoniae*. Its inception stems from studies by Todd and Hewitt on the metabolic functions of the pneumococcus (Todd and Hewitt, 1932). The earlier version of this broth constituted of a meat broth with added 2% proteose peptone (enzymatic digest of protein), diluted tenfold in a buffered salt medium, bubbled with CO_2_ to reach a pH of 6.8, and lastly supplemented with glucose (**Table 2**). They later added to this medium by supplementing with metals and phosphate (Hewitt, 1932). Hewitt’s experiments (discussed below) not only demonstrated the importance of phosphate addition to media as a buffering agent, but it was also significant for the determining metabolism of *Streptococci*. These studies culminated with the current version of Todd Hewitt broth, later streamlined by Updyke with Difco in the commercially available dehydrated version (Updyke et al., 1953) (**Table 3**).

**Table 2:** Original Todd Hewitt.

Todd Hewitt early 1932	Todd Hewitt late 1932
Meat broth 100 ml/L	Meat broth 100 ml/L
2% proteose peptone 0.2%	2% proteose peptone 20 g
(NH_4_)_2_SO_4_ 5 g/L	(NH_4_)_2_SO_4_ 5 g/L
NaHCO_3_ 4 or 10 g/L	NaHCO_3_ 8 or 10 g/L
MgSO_4_ 0.01 g/L	MgSO_4_ 0.01 g/L
Glucose 6 or 10 g/L	Glucose 5 or 10 g/L
KCl 1g/L	KCl 1g/L
	Sodium phosphate 0, 4, or 8 g/L

**Table 3:** Todd Hewitt.

BD Bacto	Millipore	Hardy Diagnostics	Himedia
Heart infusion 3.1 g/L	Beef heart infusion 10 g/L	Beef heart infusion 3.1 g/L	Beef heart infusion 10.1 g/L
Neopeptone 20 g/L	Peptic digest of animal tissue 20 g/L	Pancreatic digest of casein 10 g/L	Peptic digest of animal tissue 20 g/L
Dextrose 2 g/L	Dextrose 2 g/L	Dextrose 2 g/L	Dextrose 2 g/L
Disodium Phosphate 0.4 g/L	Disodium phosphate 0.4 g/L	Disodium phosphate 0.4 g/L	Disodium phosphate 0.4 g/L
Sodium Carbonate 2.5 g/L	Sodium Carbonate 2.5 g/L	Sodium Carbonate 2.5 g/L	Sodium Carbonate 2.5 g/L
		Yeast extract 10 g/L	
NaCl 2 g/L	NaCl 2 g/L	NaCl 2 g/L	NaCl 2 g/L

Hardy Diagnostics makes what is typically referred to as THY broth or Todd Hewitt plus yeast.

The original Todd Hewitt formulation was used to describe lactic acid formation from glucose breakdown in cultures of hemolytic *Streptococci* sp. (Todd and Hewitt, 1932). Moreover, Todd and Hewitt found that when inorganic phosphate was added to their formulation, there was a significant increase in the percentage of glucose breakdown and this effect was concentration dependent. Experiments by Mason et al., 1981 expanded on the glucose-phosphate idea. They discovered a correlation between high phosphate concentrations inhibiting pyruvate kinase in starved *Streptococcus lactis* cells (Mason et al., 1981). Starved cells returned to homeostasis after glucose addition (Mason et al., 1981).

Currently, different manufacturers of Todd Hewitt broth further adjust their formulations, for example: BD Bacto and Hardy Diagnostics add 3.1 g/L of beef heart infusion, while HiMedia adds 10.1 g/L, and Millipore includes 10 g/L (**Table 3**). Further, Hardy Diagnostics adds yeast in their powder while other formulations, it can be added separately. Additionally, the other peptone sources also differ by including enzymatic digest of milk protein or enzymatic digestion of animal tissues. Due to the known carbohydrates in peptone, and their effect on many cellular processes not limited to metabolism and capsule production, it is conceivable that these differences could affect the outcome of an experiment.

### Rane and Subarrow

Rane and Subarrow (**Table 4**) devised a defined medium to determine the nutritional requirements of at least five different serotypes (Rane and Subbarow, 1940). Rane and Subbarow substituted the complex component gelatin with a defined mixture of amino acids and other additives in their basal medium I to make basal medium II. From this new formulation, they identified choline, pantothenic acid, nicotinic acid, and amino acids arginine, histidine, and glutamic acid as essential for growth (Rane and Subbarow, 1940).

**Table 4:** Rane and Subbarow, 1940.

Component	Concentration g/L
Basal Medium I	
Acid hydrolyzed Eastman de-ashed gelatin	6.0 g
D-Glutamic acid	0.1 g
L-Cystein	0.025 g
KH_2_PO_4_	5.0 g
Basal Medium II	
D-Glutamic acid	1.0 g
Glycine	0.25 g
L-Asparagine	0.2 g
D-L Leucine	0.15 g
D-Arginine carbonate	0.075 g
D-L Alanine	0.05 g
D-L Lysine dihydrochloride	0.05 g
D-L Methionine	0.05 g
L-Cysteine	0.05 g
D-L Histidine monohydrochloride	0.025 g
L-Tryptophan	0.025 g
β-Alanine	0.025 g
Nor-Leucine	0.015 g
L-Phenylalanine	0.01 g
L-Oxyproline	0.01 g
KH_2_PO_4_	5.0 g
NaCl	2.5 g
Distilled water	800 mL
Vitamins tested	Concentrations range tested
Pantothenic acid	0-5 µg/ml
Nicotinic acid	2-50 µg/ml
Choline	0-50 µg/ml
Thioglycollic acid	0-250 µg/ml
Flavin	0-1 µg/ml

Choline is an important structural component of *Streptococcal* teichoic acid in the cell wall based on cell fractionation experiments (Mosser and Tomasz, 1970). It was also determined that choline incorporation in the cell wall was necessary for the autolytic process, as cells treated with ethanolamine (EA), which can incorporate itself into the teichoic acid, were resistant to autolysis. Briles et al. in 1970 furthered these studies and demonstrated that cells initially grown with ^3^H-labeled choline, which were later moved to EA media, resulted in cells that couldn’t separate post division, and thus formed long chains instead of the usual diplococci phenotype (Tomasz, 1968; Briles and Tomasz, 1970). Additionally, it was demonstrated that cells grown without choline were less likely to uptake DNA during transformation citing inability to respond to competence factor (Tomasz, 1968; Tomasz et al., 1971).

### Adams and Roe

Adams and Roe (**Table 5**) devised a medium to grow more fastidious pneumococcal serotypes as they were unable to subculture their isolates with the Rane and Subarrow medium and examine sulfonamide resistant bacteria (Adams and Roe, 1945). This medium is based on a 1942 recipe for growing hemolytic *Streptococci* (Bernheimer et al., 1942). It is a semi-defined medium that contains hydrolyzed casein as an undefined ingredient as meat infusion and peptones had sulfonamide inhibitors. While arginine and histidine were determined to be essential for pneumococcal growth, they were not added explicitly here. However, undefined components (e.g., casein digest) can contain as much as 4.8% arginine (Klotz et al., 2017) and 4.8% histidine (Becton-Dickinson, 2006). After this enzymatic hydrolysis, significant amounts of these amino acids are available in small and more bio-available oligopeptides. With their new medium, Adams and Roe were able to both subculture their strains and identify asparagine as crucial to sulfonamide-resistant *Streptococcus* (Adams and Roe, 1945).

**Table 5:** Adams and Roe, 1945.

Component	Concentration g/L
Basal Medium I	
Acid hydrolysate of casein	200 ml of 10% solution
L-Cysteine	150 mg
L-Tryptophan	20 mg
KCl	3.0 g
NaHPO_4_.12H_2_O	7.5 g
MgSO_4_.7H_2_O	0.5 g
Distilled Water	900 ml
Vitamin Solution	
Component	Concentration mg/100 ml
Biotin	0.015 mg
Nicotinic acid	15 mg
Pyridoxine	15 mg
Calcium pantothenate	60 mg
Thiamine	15 mg
Riboflavin	7 mg
Adenine sulfate	150 mg
Uracil	150 mg
Solution II Salt Mixture	
Component	Concentration in 100 ml
FeSO_4_.7 H_2_O	50 mg
CuSO_4_.5 H_2_O	50 mg
ZnSO_4_.7 H_2_O	50 mg
MnCl_2_.4 H_2_O	20 mg
HCl concentrated	1 ml
Add Per Liter of Solution	
Vitamin mixture solution I	8 ml
Salt mixture solution II	2 ml
20% glucose solution	10 ml
Glutamine	200 mg
Asparagine	100 mg
Choline	10 mg
CaCl_2_.2 H_2_O	10 mg
Distilled water	50 ml
Bicarbonate—thioglycollate mixture*	5%

*Prior to inoculation, add 1 mL of thioglycolic acid to 9 mL of sterile distilled water. Mix and incubate in boiling water bath for 10 minutes. Add 10 mL of sterile distilled water to 200 mg of autoclaved sodium bicarbonate. Add 200 µL of the thioglycolic acid mixture to the bicarbonate mixture.

Hoeprich would extensively modify this medium five years later: adding L-tyrosine increasing concentrations of iron, potassium, phosphate, glucose, glutamine, asparagine, uracil, adenine, choline, Vitamins B1-3, 5-7 (thiamine, riboflavin, nicotinic acid, pantothenate, pyridoxine, and biotin respectively) omitting copper and calcium; and decreasing the concentrations of zinc and manganese (Hoeprich, 1955). The addition of uracil to growth media was not pioneered by Hoeprich, as this ingredient appeared in earlier versions of Rane and Subarrow’s medium and in other groups studying *Streptococci*. Nevertheless, many of the preceding media include uracil or uridine (glycosylated uracil) as an important component.

The physiological relevance of uracil was explored by Carvalho et al. in 2013 where they demonstrated that the lack of uracil increased the lag phase of D39 but had no effect on the R6 strain (and unencapsulated derivative of D39) (Carvalho et al., 2013). Ultimately, uracil was found to be involved in pneumococcal capsule production by 1) measuring less capsule production in uracil deficient media; 2) a spontaneous D39 capsule production mutant was largely nonresponsive in growth in the presence of uracil; and 3) the native D39 *cps* promoter was 25% lower without uracil present demonstrating effects at a transcriptional level (Carvalho et al., 2018).

### Lacks and Hotchkiss

Another widely used, semi-defined media is Lacks and Hotchkiss (**Table 6**), also referred to as a modified Adams and Roe (1945). It has been the base for many modern media. This medium incorporated complex components such as yeast extract, casein hydrolysate, and BSA (Lacks and Hotchkiss, 1960). It also eliminated pure components that were deemed important to growth in previous work such as choline, arginine, histidine, and glutamic acid/glutamine. In 1966, Lacks media was revised to include: asparagine, glutamine, adenine, choline chloride, sodium bicarbonate and catalase (Lacks, 1966). *S. pneumoniae* is known to produce high levels of H_2_O_2_ but lacks catalase (Tettelin et al., 2001). In fact, the level of H_2_O_2_ that *S. pneumoniae* can make approaches low mM levels and can be detected in the breath of pneumococcal infected individuals (Majewska et al., 2004; Erttmann and Gekara, 2019).

**Table 6:** Lacks and Hotchkiss, 1960.

Component	Amount Per Liter
Casein hydrolysate	5 g
Tryptophan	6 mg
Cysteine	35 mg
Sodium acetate	2.0 g
Salts	
K_2_HPO_4_	8.5 g
MgCl_2_.6 H_2_O	0.5 g
CaCl_2_	2.5 mg
MnSO_4_.4 H_2_O	25 µg
FeSO_4_.7 H_2_O	0.5 µg
CuSO_4_.5 H_2_O	0.5 µg
ZnSO_4_.7 H_2_O	0.5 µg
Vitamins	
Biotin	0.2 µg
Nicotinic acid	0.2 mg
Pyridoxine –HCl	0.2 mg
Thiamine-HCL	0.2 mg
Riboflavin	0.1 mg
Calcium pantothenate	0.6 mg
Other	
Glucose	2 g
4% Bovine Serum Albumin	12 ml
Fresh yeast extract	30 ml

Maltose medium substituted 2 g of maltose for glucose and yeast extract replaced by 10 mg/L glutamine and 5 mg/L adenine.

### Sicard

In 1964, Sicard developed a fully synthetic (defined) medium as the others “have proven inadequate for the culture of strains used in this laboratory” (**Table 7**) (Sicard, 1964). He was working with a type 2 rough strain or Avery’s (Avery et al., 1944). While known at the time, Sicard’s work solidified that different strains of the pneumococcus varied in the media that they could grow. Sicard used either linoleic acid, spermidine, or bovine serum albumin in this new medium (see **Table 7**). Unsaturated fatty acids (such as oleic, linoleic, and linolenic acids) have been reported to improve the growth of *S. anginosus* as well as increase the production of capsule polysaccharide under low CO_2_ conditions (Matsumoto et al., 2019). Furthermore, Gullett et al. (2019) demonstrated that host polyunsaturated fatty acids are bound by a protein called FakB3 to enable the utilization of polyunsaturated fatty acids for incorporation into its membrane and therefore, minimize the metabolic expenditure of *de novo* membrane precursor synthesis (Gullett et al., 2019).

**Table 7:** Sicard, 1964.

Component	Amount Per Liter
Glucose	4.0 g
Pyruvate, Sodium	0.8 g
Uracil	1.0 mg
*Linoleic Acid	10^-7^ g
*Spermidine Phosphate	10^-5^ g
Minerals and buffer (pH = 7.55)	
NaCl	5.0 g
NH_4_Cl	2.0 g
KCl	0.4 g
MgSO_4_	0.024 g
CaCl_2_	0.010 g
FeSO_4_ 7 H_2_O	0.0055 g
Tris- (hydroxymethyl) aminomethane	4.84 g
Na_2_HPO_4_	0.12 g
FeSO_4_ 7H_2_O	0.55 mg
Riboflavin	0.3 mg
Vitamins	
Biotin	0.015 mg
Choline	5.0 mg
Nicotinamide	0.6 mg
Pantothenate, Calcium	2.4 mg
Pyridoxal HCl	0.6 mg
Riboflavin	0.1 mg
Thiamine	0.6 mg
Amino Acids	
L-Arginine	200 mg
L-Asparagine	10 mg
L-Cysteine-HCl	100 mg
L-Glutamine	20 mg
Glycine	120 mg
L-Histidine	150 mg
L-Isoleucine	6.55 mg
L-Leucine	6.55 mg
L-Lysine	420 mg
L-Methionine	180 mg
L-Threonine	175 mg
L-Valine	5.85 mg

*Can be replaced by 0.8 g of BSA fraction V.

### M17

M17 was developed in 1975 as a complex media for the improved cultivation of lactic acid bacteria particularly, lactic acid producing *Streptococci* sp. (Terzaghi and Sandine, 1975) (**Table 8**). M17 improved on its predecessor M16 with the addition of disodium glycerophosphate (GP). Phosphate was omitted in M16, relying on peptone and acetate for buffering capacity, as it would precipitate with calcium during phage assays. While having buffering capacity, GP can form a soluble complex with calcium. With this increased soluble interaction with metal, GP helped maintain the pH of the growth medium leading to improvement in cultivation as previously with some *Streptococci* sp., they had observed a pH below 6 after 24-hour growth at 30°C. Presently, different manufacturers offer a near-identical product through their websites. However, the Millipore Sigma formulation contains a combination of digested protein source (**Table 8**). Usually, but not in all cases, the product arrives without a carbon source for the investigator to provide post sterilization. The suggestion is 14.6 mM (5 g/L) of lactose; however, other sugars have also been used in the literature most commonly, glucose.

**Table 8:** M17.

Original 1975	Difco™ M17	Himedia 1029	Millipore Sigma
Polypeptone 5.0 g	Pancreatic Digest of Casein 5.0 g	Peptic digest of animal tissue 2.5 g Casein enzymic hydrolysate 2.5 g	Tryptone, 2.5 g Meat peptone (peptic), 2.5 g
Phytone peptone 5.0 g	Soy Peptone 5.0 g	Papaic digest of soyabean meal 5.0 g	Soy peptone (papainic), 5.0 g
Yeast Extract 2.5 g	Yeast Extract 2.5 g	Yeast extract 2.5 g	Yeast Extract 2.5 g
Beef Extract 5.0 g	Beef Extract 5.0 g	Beef Extract 5.0 g	Meat Extract 5.0 g
Lactose 5.0 g	Lactose 5.0 g	Lactose 5.0 g	Lactose 5.0 g
Ascorbic Acid 0.5 g	Ascorbic Acid 0.5 g	Ascorbic Acid 0.5 g	Ascorbic Acid 0.5 g
Disodium glycerophosphate 19.0 g	Disodium-β-glycerophosphate 19.0 g	Disodium glycerophosphate 19.0 g	Sodium glycerophosphate, 19.0 g
1.0 M MgSO_4_.7H_2_O 1 ml.	MgSO_4_ 0.25 g	MgSO_4_ 0.25 g	MgSO_4_ 0.25 g

Recipe is per 950 mL before adding sterile lactose solution with the exception of Himedia in which the lactose is included in the dry powder.

Tryptone is tryptic digest of casein [Trypsin cleaves peptides on the C-terminal side of lysine and arginine amino acid residues].

Peptone is enzymatic digest of animal tissues [pepsin preferentially cleaves at Phe and Leu in position P1].

### Van de Rijn and Kessler

Van de Rijn and Kessler formulated a chemically defined medium (CDM) that would yield high growth, short lag phase, and most notably, accurate virulence factor production for Group A streptococci (GAS), after previous CDMs did not support their needs in these areas (**Table 9**) (Van De Rijn and Kessler, 1980). The main purpose of obtaining this faster lag phase was to eliminate the selection and adaptation of bacterial during transfer from complex media, mostly, in reference to making M protein, for which, they were successful. This medium was also successful in that it allowed for growth small inoculum to be used with a short lag time and the doubling time was comparable to other complex media (Todd Hewitt) with a higher buffering capacity. They empirically determined this medium’s components which contained vitamins, minerals, trace metals (except zinc and copper), purines, sodium bicarbonate, and phosphates (**Table 9**).

**Table 9:** Van De Rijn and Kessler, 1980.

Component	Amount Per Liter
Metals	
FeSO_4_.7H_2_O	5.0 mg
Fe(NO_3_)_2_.9 H_2_O	1.0 mg
K_2_HPO_4_	200 mg
KH_2_PO_4_	1000 mg
MgSO_4_.7H_2_O	700 mg
MnSO_4_	5.0 mg
Amino Acids	
L-Alanine	100 mg
L-Arginine	100 mg
L-Aspartic acid	100 mg
L-Cysteine	50 mg
L-Glutamic acid	100 mg
L-Glutamine	200 mg
Glycine	100 mg
L-Histidine	100 mg
L-Isoleucine	100 mg
L-Leucine	100 mg
L-Lysine	100 mg
L-Methionine	100 mg
L-Phenylalanine	100 mg
L-Proline	100 mg
Hydroxy-L-Proline	100 mg
L-Serine	100 mg
L-Threonine	200 mg
L-Tryptophan	100 mg
L-Tyrosine	100 mg
L-Valine	100 mg
Vitamins	
p-Aminobenzoic acid	0.2 mg
Biotin	0.2 mg
Folic acid	0.8 mg
Niacinamide	1.0 mg
β-Nicotinamide adenine dinucleotide	2.5 mg
Pantothenate calcium salt	2.0 mg
Pyridoxal	1.0 mg
Pyridoxamine dihydrochloride	1.0 mg
Riboflavin	2.0 mg
Thiamine hydrochloride	1.0 mg
Vitamin B12	0.1 mg
Other	
Glucose	10,000 mg
Adenine	20 mg
Guanine hydrochloride	20 mg
Uracil	20 mg
CaCl_2_.6H_2_O	10 mg
NaC_2_H_3_O_2_.3H_2_O	4,500 mg
NaHCO_3_	2,500 mg
L- Cysteine	500 mg
NaH_2_PO_4_.H_2_O	3,195 mg
Na_2_HPO_4_.2H_2_O	7350 mg

### RPMI 1640

RPMI 1640 is a completely defined, well-known cell culture formulated at Roswell Park Memorial Institute (**Table 10**). Growth in this medium was likely adopted due to it being defined and its popularity in culturing mammalian cells, in which both (presumably) host cells and bacteria would have the same nutritional availability. RPMI was used for the cultivation of the pneumococcus, with a few modifications such as the addition of 0.4% BSA, 1% vitamin solution from Sicard, and 2 mM glutamine (Sicard, 1964; Brown et al., 2001). In 2014, Schulz devised a number of supplements suitable for the cultivation of the pneumococcus in RPMI 1640 which included 30.5 mM glucose, 0.65 mM uracil, 0.27 mM adenine, 1.1 mM glycine, 0.24 mM choline chloride, 1.7 mM NaH_2_PO_4_·H_2_O, 3.8 mM Na_2_HPO_4_, and 27 mM NaHCO_3_ (Schulz et al., 2014).

**Table 10:** RPMI 1640 Thermo.

Components	mg/L
Amino Acids	
Glycine	10
L-Arginine	200
L-Asparagine	50
L-Aspartic acid	20
L-Cystine 2HCl	65
L-Glutamic Acid	20
L-Glutamine	300
L-Histidine	15
L-Hydroxyproline	20
L-Isoleucine	50
L-Leucine	50
L-Lysine hydrochloride	40
L-Methionine	15
L-Phenylalanine	15
L-Proline	20
L-Serine	30
L-Threonine	20
L-Tryptophan	5
L-Tyrosine disodium salt dihydrate	29
L-Valine	20
Vitamins	
Biotin	0.2
Choline chloride	3
D-Calcium pantothenate	0.25
Folic Acid	1
Niacinamide	1
Para-Aminobenzoic Acid	1
Pyridoxine hydrochloride	1
Riboflavin	0.2
Thiamine hydrochloride	1
Vitamin B12	0.005
i-Inositol	35
Inorganic Salts	
Calcium nitrate (Ca(NO_3_)_2_.4H_2_O)	100
Magnesium Sulfate (MgSO_4_) (anhyd.)	48.84
Potassium Chloride (KCl)	400
Sodium Bicarbonate (NaHCO_3_)	2000
Sodium Chloride (NaCl)	6000
Sodium Phosphate dibasic (Na_2_HPO_4_)	800
Other Components	
D-Glucose (Dextrose)	2000
Glutathione (reduced)	1
Supplements from Schulz 2014	Final concentration (mM)
Glucose	30.5
Uracil	0.65
Adenine	0.27
Glycine	1.1
Choline chloride	0.24
NaH_2_PO_4_	1.7
Na_2_HPO_4_	3.8
NaHCO_3_	27

### Host-Like or Niche-Specific Media

For early clinical microbiology, a rate-limiting step was consistent isolation and propagation. Understandably, early culture media were nutrient-rich to ensure replication of the inoculum for differential diagnosis or experimentation. We will later highlight that pneumococcal growth rates, gene transcription, protein expression, and virulence (including the capsule) are known to vary by media type and O_2_ concentration. However, these rich media were not designed to reflect the abundance (or chemical form) of nutrients *in vivo*. A prime example is the choice of carbon source, or fermentable sugar, where glucose is often used. *S. pneumoniae* grows fast and vigorous in glucose, but rarely encounters this sugar except in the blood and cerebrospinal fluid (CSF) niches. The host-pathogen interface is a critical area of pneumococcal research and several studies have developed or used media intended to replicate conditions within the hosts, especially given the range of niches (e.g., nasopharynx and pulmonary epithelia, blood, CSF) in which *S. pneumoniae* persists.

It has been shown that *S. pneumoniae* grows equally fast in minimal media supplemented with porcine gastric mucin versus culture in BHI (Yesilkaya et al., 2008). Mucins are large proteins, extremely abundant in the inner walls of respiratory epithelia, and heavily glycosylated leading to molecular weights in mega-Dalton range. Despite a relatively small genome, *S. pneumoniae* encodes dozens of deglycosylases, proteases, and importers which enable the pathogen to turn an innate immune barrier (mucous) into a buffet (Bidossi et al., 2012; Buckwalter and King, 2012).

Most recently, van Beek et al. explored metal availability in the host nasopharynx by measuring the levels of free and protein bound metals in human nasal fluid of healthy individuals (van Beek et al., 2020). When comparing their CDM as defined by the Kloosterman et al. paper for “extending the molecular toolbox” of the pneumococcus against nasal fluid, it was found that nasal fluid contained higher levels of calcium (~10x), magnesium (~1.5x), copper (~10x), and iron (~12x) while having lower concentrations of manganese (>250x), cobalt (>15x), and zinc (~1.5x) (Kloosterman et al., 2006; van Beek et al., 2020). Of note, most of the iron, copper, and zinc in the nasal fluid were protein-bound and not free. To better mimic the host environment, van Beek et al. made an *in vivo* mimicking CDM, termed IVM-CDM, that more closely resembled the nasal fluid with no metal concentration differing by more than 20% (van Beek et al., 2020). This host-pathogen interface is of particular interest in pneumococcal colonization and by creating a chemical defined media containing similar concentrations of metals available in the nasopharynx, van Beek et al. was able to bring the field closer to a recipe that truly resembles the host niche.

Using the IVM-CDM, van Beek et al. also highlighted nine proteins that were surface exposed, conserved, most part of the core genome (8/9), and expressed in increased abundance during colonization as potential vaccine antigens. Five of these proteins induced antibody responses in mice—PsaA (Pneumococcal surface adhesin A), MetQ (D-methionine-binding lipoprotein), AdcAII (zinc-binding lipoprotein), PrtA (serine protease, and AliA (Oligopeptide-binding protein)—with AliA having the most protective effect as measured by reduced bacterial load (van Beek et al., 2020). As such, growing bacteria in media mimicking their native environment here and detailed in other protocols (Suárez and Texeira, 2019) is an effective method in the quest to make new treatments and vaccines.

### Blood Agar Plates

One of the benefits of growing *S. pneumoniae* in blood agar plates is their distinctive colony morphology and alpha-hemolytic characteristic which quickly aids in its identification. However, even this simple method isn’t standardized among investigators. Two commonly used bases for blood agar plates are tryptic soy agar or Columbia agar (**Tables 11** and **12**). These base media also differ in their composition, with Columbia agar having a wider source of amino acids as well as the addition of corn starch (Ellner and Stoessel, 1966), which enhances hemolytic reactions as well as marking differences in colony morphology between *Streptococcal* spp. The most used blood sources are defibrinated 3-10% (5% being most common) sheep or horse blood, but rabbit blood has also been used as an agar base. Mammalian blood is a poorly defined media supplement as it contains tens of thousands of protein isotypes in various states (e.g., phosphorylated, glycosylated, lipidated). This is especially true with lysed blood.

**Table 11:** Tryptic soy agar Criterion™.

Component	g/L
Pancreatic Digest of Casein	15.0 g
Peptic Digest of Soybean Meal	5.0 g
Sodium Chloride	5.0 g
Agar	15.0 g

**Table 12:** Columbia agar Criterion™.

Component	g/L
Pancreatic Digest of Casein	12.0 g
Peptic Digest of Animal Tissue	5.0 g
Sodium Chloride	5.0 g
Yeast Extract	3.0 g
Beef Extract	3.0 g
Corn Starch	1.0 g
Agar	11.0 g

Early comparisons between the species’ blood (with or without cell lysis) in culture media demonstrated that the sensitivity of bacteria to sulfonamide and diaminopyridine. Ferone et al. reasoned that this effect of lower effectiveness was due to an excess of a supplemental nutrient (Ferone et al., 1975). It was important to remove the source of inhibition—i.e., the active factor(s) in the blood—using solid Mueller-Hinton Broth (MHB) plates. To test their hypothesis, they supplemented this media with a downstream product, thymidine, which requires folate metabolism. By supplying this downstream metabolite, any drug targeting folate or nucleotide synthesis would have little effect. Ferone et al. found that horse and human blood contain thymidine phosphorylase (absent in sheep’s blood), also known as Harper-Cawston factor (Harper and Cawston, 1945). This protein phosphorylates, then cleaves thymidine into thymine and 2-deoxyribose-1-phosphate. Naturally, some bacteria are less efficient using thymine over thymidine, which led to results that varied by the species of blood used. It is likely that abundant and resilient proteins (e.g. catalase) retain substantial activity—despite the heat shock of mixing with liquid agar—and interact with media nutrients and bacterial metabolites as small molecules diffuse through the agar matrix demonstrated in antimicrobial disk diffusion assays. However, little is known regarding the persistence and dynamics of blood protein biochemistry in agar or co-culture with pathogens.

### Anaerobic Growth

Pneumococcus is habitually cultured as an aerobe with 5% CO_2_ atmosphere, where it seems to thrive despite lacking catalase or cytochromes. However, it is a facultative anaerobe. Many different methods have been used to produce hypoxic environments such as Gas-Pak^®^ systems, anaerobic chambers, and oxygen scavengers. Anoxic growth has been previously reported to facilitate the isolation of clinical samples. Howden used an atmosphere of 90% hydrogen and 10% CO_2_ to facilitate the isolation of clinical isolates (Howden, 1976). Four years later Wu et al. compared three methods for the recovery of *Streptococcus* from lower respiratory secretions and found that using a Gas-Pak^®^ allowed them to recover 93% of their clinical specimens (Wu et al., 1980). The increased isolation was hypothesized to be due to a shortened lag phase during anaerobic growth and a reduction in autolysin production under anaerobic conditions (Howden, 1976; Nagaoka et al., 2019). However, conversely, Baesman and Strand (1984) compared isolation using aerobic and anaerobic methods (a Bio-Bag Environmental Chamber A anaerobic system), observing no significant difference between either (Baesman and Strand, 1984).

Despite the hundreds of isolates gathered across these three studies, none of them mentioned the serotype for any isolate. While the CDC published a widely used isolate culture method using broth enrichment (Todd Hewitt broth supplemented with 1 mL of rabbit serum), followed by real-time PCR of *lytA* (pneumococcal autolysin) for identification in 2010 (da Gloria Carvalho et al., 2010), Tothpal et al. performed a major survey of 256 pneumococcal strains under different experimental conditions including temperature and oxygen availability while including typing (Tothpal et al., 2019). For an anaerobic environment, they utilized the oxygen scavenger Oxyrase^®^. This study showed that there was significant variation between the serotype growth in anaerobic and aerobic environments, and variations thereof—such as serotype 4— which grew to higher optical density when cultured aerobically with catalase than anaerobically (Tothpal et al., 2019). In spite of ambient O_2_, catalase use in the aerobic growth media supported higher maximum growth. Therefore, culture density for some serotypes is linked to the flux and concentration of H_2_O_2_ (in the presence of glucose), and was determined by capsule switching experiments, not linked to the capsular type alone.

While this is not an exhaustive list of all pneumococcus solid and liquid media, it covers most reported growth media. Considering *S. pneumoniae* is genetically diverse with an estimate of 50% core genome at the species level, there is a strong possibility that variations in the nutritional value of these media can have an impact on bacterial physiology (Donati et al., 2010).

## Media Matters: Effects of Media on Gene Expression and Metabolism

There are many nuances to microbial culture media, especially those reported for growing *S. pneumoniae*. It is evident that these media can vary in amino acid composition, abundance of vitamins, metals, carbon source availability, and more. In the previous sections, we described how different media was developed to grow fastidious pneumococcal serotypes (to higher OD and with shorter lag time) and mimic the host environment. Below we present specific examples of how media can change capsule dynamics, gene expression, metabolism, protein profiles, and antibiotic susceptibility. Ultimately, studies rarely focus on comparing microbial behavior at the genetic, proteomic, and metabolomic level in different media, thus this is a limitation in finding examples.

### Antibiotic Susceptibility

While the host makes itself non-hospitable to pathogenic bacteria, sometimes it needs assistance in the form of an antibiotic. Bacteria are quite adaptable in forming resistance to these drugs by mutating the target of the antimicrobial compound or by making efflux pumps to export them. It is important to have a standardized measure of bacterial susceptibly to a given antibiotic. Antimicrobial susceptibility testing (AST) is done *in vitro* using media like blood agar plates usually with the base of Mueller-Hinton Broth (MHB), a rich medium, to find minimum inhibitory concentration (MIC) (Clinical and Laboratory Standards Institute, 2012). For the pneumococcus, the standard AST procedure as defined by the Clinical and Laboratory Standards Institute (CLSI) uses cation adjusted MHB (calcium at 20 to 25 mg/L and magnesium at 10 to 12.5 mg/L) with 2.5-5% lysed horse blood (Clinical and Laboratory Standards Institute, 2012). AST is critical in tracking geographic and phenotypic changes over time in global antibiotic resistance. When developing candidate antibiotics, this same assay is used to establish the feasibility and capability of a hit compound. Given what we have briefly reviewed on media design and use, such a rich media may not be suitable and may be a limitation of these assays.

While several laboratories have investigated AST in other organisms (Rajput et al., 2019; Salazar et al., 2020), Ersoy et al. in 2017 looked at the pneumococcus as a bacterium of study (Ersoy et al., 2017). In comparing these effects of host-like media and other complex media on antibiotic resistance, the MICs of some drugs varied by at least a factor of 4 (Ersoy et al., 2017). Overall, Ersoy et al. found that the standard AST of MHB frequently failed to adequately identify antibiotic susceptibility *in vivo*. They also provide evidence that host-like media could give be more predictive of MIC *in vivo* (Ersoy et al., 2017). In one example, the folate-targeting drug trimethoprim was shown to be effective against most tested serotypes of *S. pneumoniae* using standard AST as defined by the CLSI, but ineffective in the host-like conditions Lacks and modified Lacks medium (Clinical and Laboratory Standards Institute, 2012). Following up, mice were infected with *S. pneumoniae* and treated with trimethoprim, which failed to protect any of the ten mice. In this case, and in many others that they tested, the standard MIC test did not accurately predict the drug’s efficacy, but the host-like conditions did. These measurements extended themselves to different serotypes demonstrating that two serotypes (6 and 23) were more resistant to ampicillin and ceftriaxone with the D39 strain exhibiting less resistance to azithromycin and erythromycin. Ersoy et al. also examined the influence of NaHCO_3_ concentration on resistance finding that as a trend adding physiological levels of sodium bicarbonate to MHB improved the *in vivo* prediction (Ersoy et al., 2017).

While conclusions regarding resistance and susceptibility differ from those obtained by CLSI methods, and thus must be looked at with scrutiny, it is nevertheless striking, but not surprising that host-like media give more reliable results than complex media. Especially in the search for new antibacterial mechanisms, standard AST media may obfuscate the effects of an otherwise promising drug. The MIC of any drug against a target organism not just a function of genome but can be strongly impacted by changes in the environment. It is further complicated by the dynamics of the other bacteria in the environment which could convey unexpected resistance (Sorg et al., 2016). The nutritional environment is a dynamic and strong factor in the laboratory, but especially at the host-pathogen interface.

### Capsule

*S. pneumoniae* dedicates 30% of its membrane transport systems to carbohydrates as it is their only source of energy (Buckwalter and King, 2012). Therefore, it is not far-fetched to imagine that quantity and type of carbon source is linked to gene expression. An example of how carbon sources can impact gene expression is illustrated by Troxler et al. (2019). This work investigated capsule thickness using FITC dextran exclusion assay for serotype 6B and 7F under different carbon sources (glucose, fructose, and sucrose) on CDM, which here was modified Lacks medium with single carbon sources added. They found a significant decrease in capsule thickness when cultured on fructose as the only carbon source and an increase in capsule thickness when grown on glucose or sucrose as confirmed by transmission electron microscopy (TEM). The capsule was mostly generated by the glucose carbons while the fructose carbons were metabolized mostly for glycolysis. Additionally, they tested intracellular metabolites from all three carbon sources using ^31^P labeled NMR and confirmed the accumulation of capsule precursors made from each carbon source in a capsule knockout model. Finally, gene expression was studied using RNA-seq. This revealed changes in the peptidoglycan synthesis genes *glmS* and *nagA* as well as pyrimidine synthesis genes changing in a carbon source dependent manner, indicating an important role for carbohydrate sources in nutrient restricted media. This point is especially poignant in the host where these sources are limited and therefore affect capsule size. Capsule size has a negative correlation with binding to luminal mucus and positive correlation with spreading to other host surfaces (Nelson et al., 2007).

Hathaway et al. tested pneumococcal mutants that could switch their capsules between two different types to determine if serotype was an important factor for growth and carriage of pneumococcus (Hathaway et al., 2012). This paper found that making different capsules is energetically costly and as such, available nutrients affect colonization in terms of long-term vs invasiveness, and growth (Hathaway et al., 2012). Inadvertently, this study found that capsule thickness increased when the pneumococcus was cultivated in rich media such as brain heart infusion with fetal calf serum as compared to cultivation in minimal media (Hathaway et al., 2012). Taken together, these studies imply that the choice and components of media directly impact capsule production and therefore can influence measures of infection.

Co-infections can also affect how the pneumococcus obtains and processes carbohydrates and morphology. This is most notably manifested in its co-infections with influenza and *Haemophlius influenzae*. Viral influenza infections increase pneumococcal access to sialic acid in the nasopharynx (Siegel et al., 2014). Sialic acid, which in addition to its ability to be a sole carbon source can also be a major component in pneumococcal capsule (Charland et al., 1995; Marion et al., 2011). During long term co-culture (more than 10 days) with *H. influenzae*, as without *H. influenzae* there was no pneumococcal growth, glucose and lactose produced varying outcomes in culturability with the latter causing a morphology change (small colony variant) and reduce capsule size (Tikhomirova et al., 2018). Co-infection between these two pathogens can also lead to increased pneumococcal biofilm production, however, resulting in decreased infection (Weimer et al., 2011).

### Transcriptomics and Proteomics

The availability of resources causes many binary changes leading to a cascade of effects at the genetic level. In an ambitious study, Aprianto et al. categorized the transcriptomes of the pneumococcus by mimicking various host environments and made a graphical user interface (https://veeninglab.com/pneumoexpress) that allows the end user to see the different expression profiles (Aprianto et al., 2018). To achieve the 22 conditions that mimicked various stages and types of infection, they varied sugar type and concentration, protein, CO_2_, temperature, acidity, and presence of epithelial cells selecting Sicard’s defined medium as the backbone of many of the conditions (Sicard, 1964; Aprianto et al., 2018). This study was notable due to the few comprehensive environment transcriptome databases that exist for model bacteria. The authors state that one limitation is that they did not vary metal ions which also play a large role in infection, however, above, we detailed the effects of metals in finding vaccine candidates (van Beek et al., 2020). Aprianto et al. found that there were 498 conditionally expressed genes based on the media conditions with ~10% of these genes involved in regulating carbohydrate import (many of which are under the control of CcpA (catabolite control protein A). Further, they found that growth in C+Y (not to be confused with CY media ATCC MD-2906) had a vastly different transcription profile than those related to infection or competence but was similar to other conditions like transmission indicating that the response was not completely tied to the media (Aprianto et al., 2018). Mining this data set will occur for years to come, likely finding more differences in how the pneumococcus behaves in various environments.

Another example of additives impacting phenotypes is documented by Ferrándiz et al. (2019). In this work, the authors were characterizing stress proteins in nutrient restricted conditions in semisynthetic AGCH (Adenine Glutamine Casein Hydrolysate) medium also known as modified Lacks media (Ottolenghi and Hotchkiss, 1962), in comparison to effects on nutrient rich conditions (Todd-Hewitt broth + yeast extract) (Ferrándiz et al., 2019). They found that when the general stress gene *gls24B* was knocked out, the mutant was able to grow only in the presence of BSA and growth was concentration dependent. While unable to determine what about the BSA was responsible for the phenotype, the effect was also observed using protease-treated BSA, but not with acid-digested BSA (Ferrándiz et al., 2019). Interestingly, BSA binds fatty acids which still need to be removed and fatty acid metabolism was downregulated in the mutant (Davis and Dubos, 1947; Chen, 1967).

In 2009, Pandya et al. used a combination of microarray and RT-PCR to study the transcriptional profile of a type 4 strain serially cultured on two broths (Pandya et al., 2009). As a control, mRNA from *S. pneumoniae* grown in Todd-Hewitt yeast extract broth was compared to the same strain cultivated 50-100 passages on blood agar plates (Pandya et al., 2009). They found that selection on blood agar increased the number of differentially expressed genes from 113 genes after 50 passages to 706 genes (or approximately 1/3 of the pneumococcus genome) after 100 passages. Of the genes most differentially expressed, roughly a third of the genes (160) were annotated as hypothetical, 60 genes of unknown function, and 93 virulence genes—including the capsule locus. Consistent with previous reports, genes associated with adhesion and colonization (e.g., neuraminidase and choline binding proteins) were downregulated during blood agar cultivation (Gosink et al., 2000; Brittan et al., 2012). While neuraminidase and choline binding proteins support bacterial colonization and dissemination respectively, neuraminidase cleaves sialic acids in the blood which recruits host complement (Attali et al., 2008; Syed et al., 2019).

These transcriptional changes highlight what occurs when the pneumococcus transitions to other environments in the host such as the blood. Bae et al. cultured D39 on Todd Hewitt agar ± 5% sheep blood and documented a more general shift in protein expression by using 2-D gel electrophoresis and matrix assisted laser desorption ionization-time of flight mass spectrometry (MALDI-TOF) spectra (Bae et al., 2006). One major change they saw with added blood was with metabolism. Lactate oxidase (*lctO*), which catalyzes the oxidation of lactate and O_2_ to pyruvate and H_2_O_2_, had lower expression (3.5 fold), L-lactate dehydrogenase (*ldh*), which reversibly converts pyruvate and NADH (nicotinamide adenine dinucleotide with hydrogen) to lactate and NAD^+^, had higher expression (4.2 fold), and pyruvate oxidase (*spxB*), which converts pyruvate, phosphate and O_2_ to acetyl phosphate and H_2_O_2_ was observed as a spot, but not increased (Bae et al., 2006). These changes mimic bacterial transition to the anaerobic conditions of the blood, an environment where *ldh* has been found to be essential (Gaspar et al., 2014). Both *spxB* (the major contributor of and making H_2_O_2_) and *lcto* are upregulated limited aeration conditions compared to anaerobic growth in BHI (Echlin et al., 2016; Lisher et al., 2017). Interestingly, it is H_2_O_2_ made by SpxB and LctO that causes α-hemolysis in blood (McDevitt et al., 2020).

Zinc import mutants grown on zinc restrictive media demonstrate morphological defects as revealed by bright field microscopy and cryo-electron microscopy (Bayle et al., 2011). These mutants were also unable to colonize a mouse model 48 hours after In or IP inoculation, indicating reduced virulence (Bayle et al., 2011). Carbon source can also play a role in how the pneumococcus maintains metal homeostasis. In fact, the addition of hemoglobin has been shown to stimulate pneumococcal usage of host carbohydrates the essentially helping it to adapt to the host environment (Akhter et al., 2020). Lack of iron also affects the pneumococcus, which produces no known siderophores and thus, must scavenge it *via* hemoglobin, heme, hemin, or take in free iron (uncommon in the host), by hindering virulence and causing dysbiosis in metabolism (Tai et al., 1993; Akhter et al., 2020). Lastly, Afzal et al. demonstrated in D39 that ascorbic acid as the carbon source in M17 led to decrease in intracellular zinc, manganese and iron (Afzal et al., 2015).

Finally, one of the most striking examples of the effects of media on bacterial physiology was illustrated by Hoyer et al. (2018). In this work, an unencapsulated variant of pneumococcus was grown on chemically defined media (RPMI 1640 & supplements) and complex media (Todd-Hewitt) under iron limiting conditions and control. Proteomic analysis was performed comparing gene expression between growth mediums. They found that 43.7% of pneumococcal proteins were uniquely expressed in one of the two mediums. This study highlights how varying a single nutrient impact nearly half of the organisms’ proteins. They also noted that many proteins involved in pathogenesis were downregulated in nutrient-restricted conditions. Among the genes differentially expressed was the iron acquisition gene *piuA* (SPD_1652) which was highly expressed under CDM vs. rich medium. Morphological differences were also revealed using scanning electron microscopy. Denser DNA was noted by TEM under nutrient restricted conditions, suggesting a decrease in active transcription. Finally, a decrease in ribosomal proteins detected *via* LC–MS/MS under CDM conditions indicating a slower growth rate.

## Suggestions and Conclusions

Throughout this process, the authors encountered numerous reports of papers using “CDM” as their bacterial media. However, as we have seen throughout this process, not all CDMs are the same or created equal. Further, in many cases, primary sources of the media were not cited, but instead, had 3-4 degrees of separation. While we understand that stating “as described in x et al.” can save on space, due to the variability in media, we strongly suggest listing the recipe for the CDM used in the paper in the supplemental text, especially if modifications were made (along with listing why modifications were made) or at the very least, citing the primary source paper containing the recipe in addition to the original method. Further, choice of media is often taken as given — based on training, tradition, or studies read and, in many cases, the reasons behind said choice of media are not given. As such, we encourage for the reasoning behind using a given medium be explained in the text.

As discussed, the media choice for *S. pneumoniae* dramatically impacts experimental outcomes. The host-pathogen interface is a critical area of pneumococcal research and several studies have developed or used media intended to replicate conditions within the host. Survival in the disparate niches (e.g., nasopharynx and pulmonary epithelia, blood, cerebrospinal fluid) in which *S. pneumoniae* persist—this is no simple task and a testament to this organism’s adaptation. Based on varying media components, it seems possible that many experimental outcomes may have been and could still be accidentally influenced by decisions regarding media. As we illustrated here, the choice of media matters for far more than organism growth and propagation. We sincerely hope that in choosing pneumococcal and all other bacterial media, that the examples provided above will be considered to enhance future research studies.

## Author Contributions

YS-R and MJ conceptualized, wrote, and edited this manuscript. All authors contributed to the article and approved the submitted version.

## Funding

NIGMS 1R35128653.

## Conflict of Interest

The authors declare that the research was conducted in the absence of any commercial or financial relationships that could be construed as a potential conflict of interest.
